# Rapid and Simultaneous *In Situ* Assessment of Aflatoxins and Stilbenes Using Silica Plate Imprinting Mass Spectrometry Imaging

**DOI:** 10.1371/journal.pone.0090901

**Published:** 2014-03-04

**Authors:** Diogo N. de Oliveira, Mônica S. Ferreira, Rodrigo R. Catharino

**Affiliations:** INNOVARE Biomarkers Laboratory, School of Medical Sciences, State University of Campinas, UNICAMP, Campinas, Brazil; Moffitt Cancer Center, United States of America

## Abstract

A fast and direct combination of techniques for simultaneous mycotoxin and phytoalexin identification in peanut skin and kernel is described. Silica Plate Imprinting Laser Desorption/Ionization Mass Spectrometry Imaging (SPILDI-MSI) is a powerful technique that exhibits great advantages, such as solvent-free and matrix-free characteristics, as well as no sample preparation or separation steps. It also permits accurate identification of mycotoxins and phytoalexins with unique fingerprint profiles in just a few seconds. Results are expressed as chemical images of the 4 identified types of aflatoxins (B1, B2, G1 and G2) and a stilbenoid (resveratrol). Also, SPILDI-MSI allows the comparison between the spatial distribution of aflatoxins and resveratrol found in kernel and skin. This novel application has proven to be useful for instantaneous qualitative assessment of aflatoxins and stilbenoids both in the peanut skin and kernel and offers precise tracking of fungal contamination in nuts and other foodstuffs.

## Introduction

Mycotoxins have been more closely monitored in the past decades due to their harsh effects observed in humans and animals; potent toxic effects in humans and animals have been related to these molecules, such as cytotoxicity, carcinogenicity, mutagenicity, neurotoxicity, hepatotoxicity, immunosuppressive, and estrogenic effects [1]–[4]. As to their occurrence, aflatoxins and ochratoxins are produced mainly by *Aspergillus* sp., fumonisins, trichothecenes and zearalenone by *Fusarium* sp., patulin by *Penicillium* sp., and ergot alkaloids, produced in the sclerotia of *Claviceps* sp. [5]. Furthermore, these compounds have great financial impact. From an economic point of view, mycotoxins cause money loss to producers, processors and also consumers of food and feeds. Significant reduction in foreign exchange is also an issue, as exported products are rejected in other countries due to the presence of these molecules [5]–[7]. In peanuts (*Arachis hypogaea* L.), *Aspergillus* sp. correspond to the main class of fungi that are associated to aflatoxin contamination, producing the types B1, B2, G1 and G2 [8].

Phytoalexins, more specifically stilbenoids, are molecules that help monitor fungal contamination [9]. They are secondary metabolites of nuts, produced in response to infections, injuries and/or other suffered attacks [10]. Many of these species are oxidation products derived from resveratrol, a phenolic compound that exhibits great antioxidant potential, especially in humans, with many potential applications for the treatment of several diseases such as cancer and cardiopathies in the past few years [11]–[14]. In plants, it is believed that an increased phytoalexin production is directly related to the defensive response of the vegetable, and this may also correspond to lower levels of aflatoxins [10].

Traditional analytical methods for assessing mycotoxins and phytoalexins include many steps of sample preparation, as liquid-liquid extraction (LLE), supercritical fluid extraction (SFE), solid phase extraction (SPE) and solid phase microextraction (SPME) [10], [15]–[21]. After these procedures, the sample is then subjected to a separation and detection system for identification and/or quantification. Generally, thin-layer chromatography (TLC), high-pressure liquid chromatography (HPLC), gas chromatography (GC) and liquid chromatography (LC) coupled to mass spectrometry (MS) detector are the most used approaches [11], [17], [22]–[26]. For these time-consuming characteristics, faster and more effective methods for high-throughput screening of mycotoxins and stilbenes in foodstuffs are necessary.

New approaches have already been developed in this field. Matrix-assisted laser desorption/ionization (MALDI) coupled with Time-of-Flight (TOF) analyzer has been successfully employed in aflatoxin screening [27]. This technique uses an energy-absorbent molecule (matrix), which is mixed with the sample or applied directly over it to assist laser ionization. Due to their characteristic structure, stilbenes have also been employed as MALDI matrices [28]. Within the most common configurations, apart from MALDI-TOF, there has recently been an increasing interest in instruments with Mass Spectrometry Imaging (MSI) [29]. This modern and interesting approach provides spatial distribution of compounds with intensities of a given ion on a coordinate system and its relative position in a physical sample, creating a sample image based on the specific molecular information measured [29].

Another recent analytical trend is the sorptive tape extraction (STE), in which a sorbent surface is used for molecular imprisonment and posterior instrumental analysis [30]. This technique requires little sample preparation and no derivatization or liquid extractions. The STE principle was used as the basis for our procedure of sample preparation for subsequent LDI-MSI analysis, where a silica gel plate for TLC was used as a sorptive tape-like support for the imprinting of samples, in a slightly modified methodology as the ones described in previous works [31], [32].

The aim of this work is to provide, for the first time, a new method for direct and simultaneous screening of aflatoxins and a stilbenoid (resveratrol) in peanuts (*Arachis hypogaea* L.) skin and kernel using SPI as a sorptive tape-like extraction method followed by LDI-MSI. A silica gel (60 Å) plate is used as a molecular trapping surface for the samples. The greatest advantage associated to these methods is that they do not require chromatographic separation or many steps of sample preparation. This is also the first work that assesses both health hazardous and beneficial compounds to humans in a single sample, at the same time.

## Materials and Methods

### Reagents and Standards

Methanol and acetonitrile were HPLC grade (>98%), purchased from J.T. Baker (Xastoloc, Mexico). Aflatoxins and resveratrol standards were purchased from Sigma-Aldrich Co. (St. Louis, MO, USA).

### Peanut samples

Commercially available raw peanut bags were purchased from grocery stores in Campinas, Brazil. The bags were properly stored in a cabinet, free from light and at 25°C. Samples were utilized after 1 year from the expiration date.

### Sample preparation

**Figure 1:**
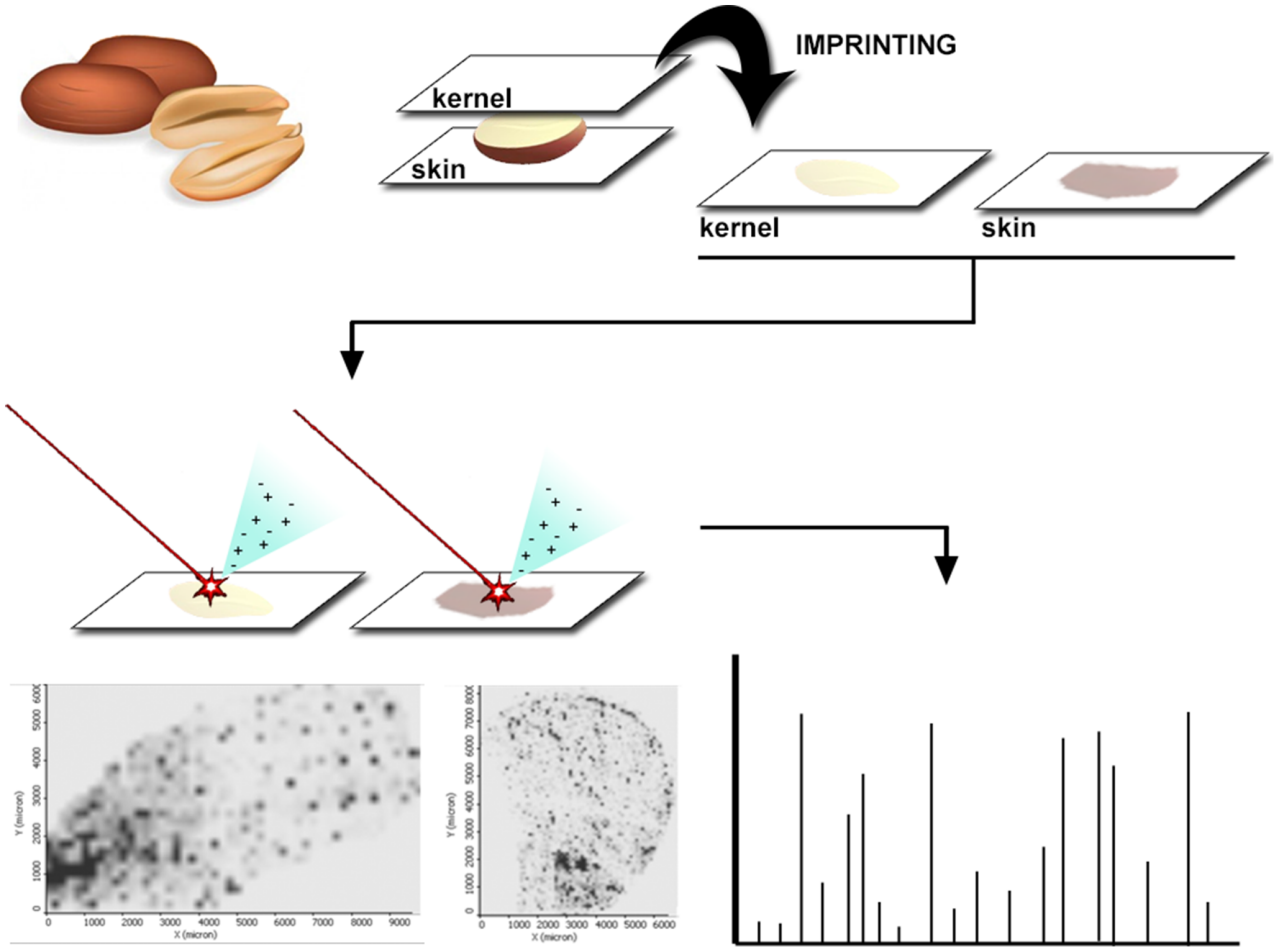
Detailed workflow of the SPILDI-MSI experiments for compound identification in peanut skin and kernel. Cross-sections of the kernel and the skin are imprinted in a TLC plate and then sent for MSI analysis.

Skin was removed from kernel and thin transversal sections of peanuts were cut with a stainless steel blade to obtain thin slices (∼1 mm) of the sample. SPILDI experiments were carried out by pressing the samples against two silica 60 TLC plates (Merck, Darmstadt, Germany) for five minutes. Preliminary tests with 1, 5, 10 and 15 minutes were performed; no signal improvements were observed with pressing times higher than 5 minutes (data not shown). Plates were then sent to analysis with no matrix coating. A representation of the analytical workflow is depicted in Figure 1. Standards were prepared as 1 mg/mL solutions in MeOH:H_2_O (50∶50). 2 μL of each standard solution were directly spotted in the TLC plate and then sent to analysis under the same MS conditions as the samples.

### Mass spectrometry imaging

Samples were analyzed in a MALDI-LTQ-XL instrument (Thermo Scientific, California, USA) with imaging feature. The instrument uses an ultraviolet nitrogen laser. Typical conditions for data acquisition were as follows: 20 μJ laser power, 100 μm raster step size with laser spot size of 50 μm (factory default setting) and 30–50 normalized collision energy for collision-induced dissociation (CID) when performing MS/MS reactions. All mycotoxins data were acquired in the positive ion mode and resveratrol was analyzed in the negative ion mode (both at the *m/z* range of 100–500).

### Data workup

The obtained MS/MS spectral data from standards and samples were submitted to structural analysis with Mass Frontier software (v. 6.0, Thermo Scientific, California, USA). The inputted structures are analyzed using algorithms and database information to produce fragment possibilities, which are then compared to the MS/MS spectra to assist in compound identification. Chemical images were treated with ImageQuest software (Thermo Scientific, California, USA) and all intensities were normalized according to the total ion current.

## Results

**Figure 2:**
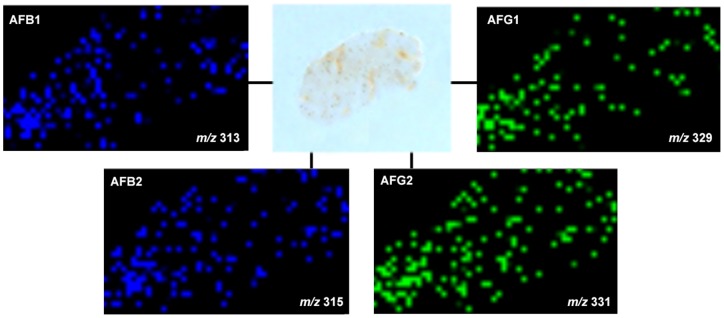
Example of SPILDI-Mass spectrometry images of the peanut skin: aflatoxins B1, B2, G1 and G2 are noted in their characteristic spatial distributions. Positive ion mode.

As the experiments were conducted with the skin and the kernel of peanuts, the spatial distribution of the different aflatoxins are compared in both regions as chemical images in Figures 2 (skin) and 3 (kernel). It was possible to observe that all types of aflatoxins were present even deeply into the internal regions of the kernel. CID was performed for the [M+H]^+^ species for identification of the different aflatoxin types, with MS/MS data presented in Figures 4 and 5 and also organized in Table 1. These data were analyzed using Mass Frontier software for fragmentation processes; they were also supported by the comparison with the MS/MS fragmentation pattern of the standards, as seen in Figure S1. Two-dimensional distributions on the surfaces of skin and kernel were collected directly via MS/MS of the characterized [M+H]^+^ species, with results plotted as follows: Aflatoxin B1 (AFB1, *m/z* 313), Aflatoxin B2 (AFB2, *m/z* 315), Aflatoxin G1 (AFG1, *m/z* 329) and Aflatoxin G2 (AFG2, *m/z* 331).

**Figure 3:**
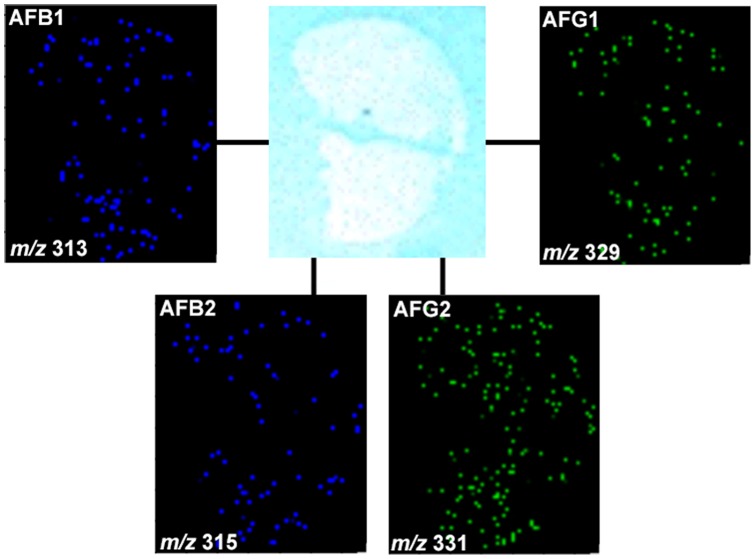
Sample SPILDI-Mass spectrometry images of the peanut kernel: aflatoxins B1, B2, G1 and G2 are noted in their characteristic spatial distributions. Positive ion mode.

**Figure 4:**
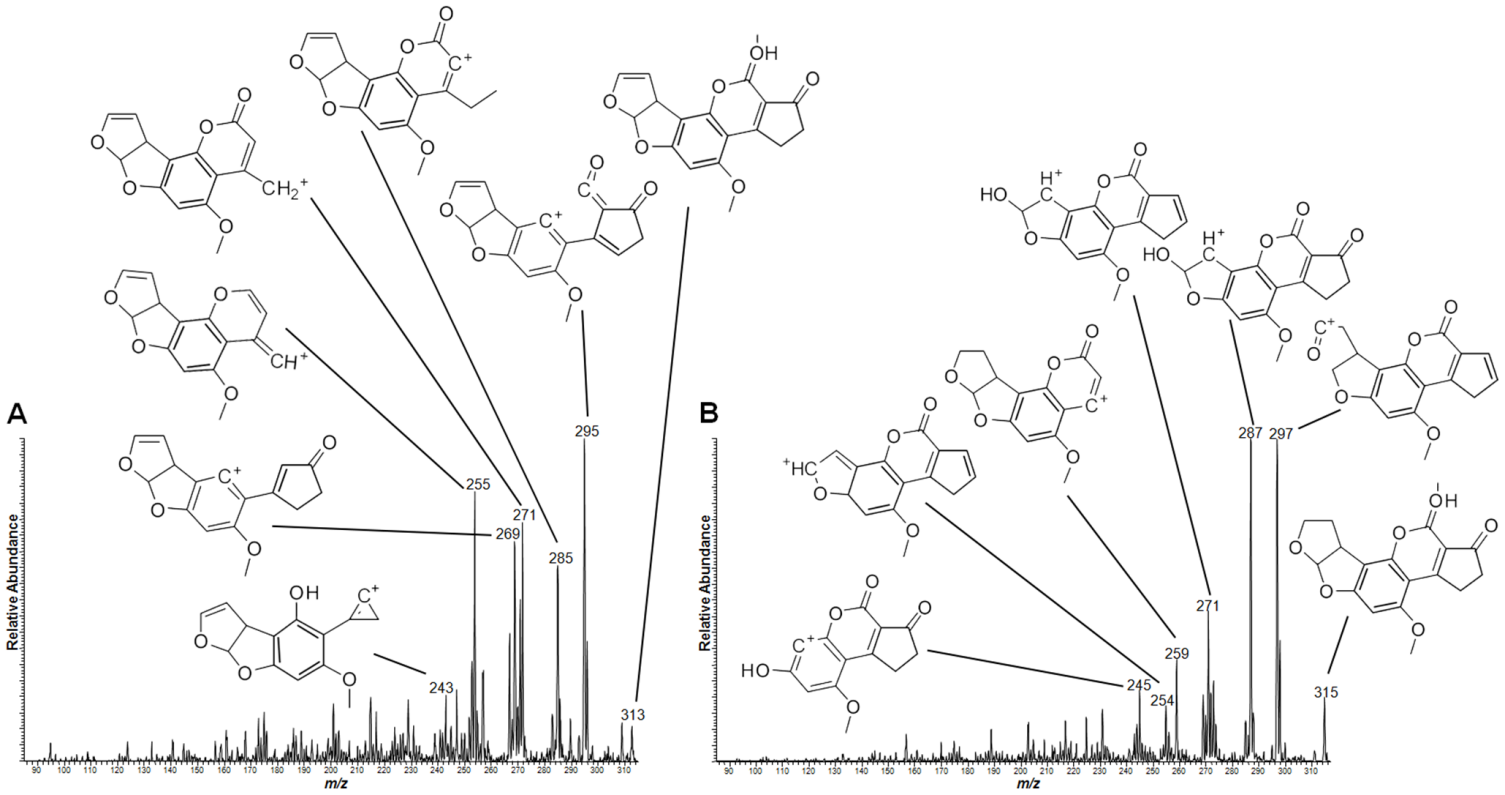
MS/MS spectra of aflatoxins (A) B1 and (B) B2. The characteristic fragments identified with Mass Frontier are identified along with the respective signals. Positive ion mode.

**Figure 5:**
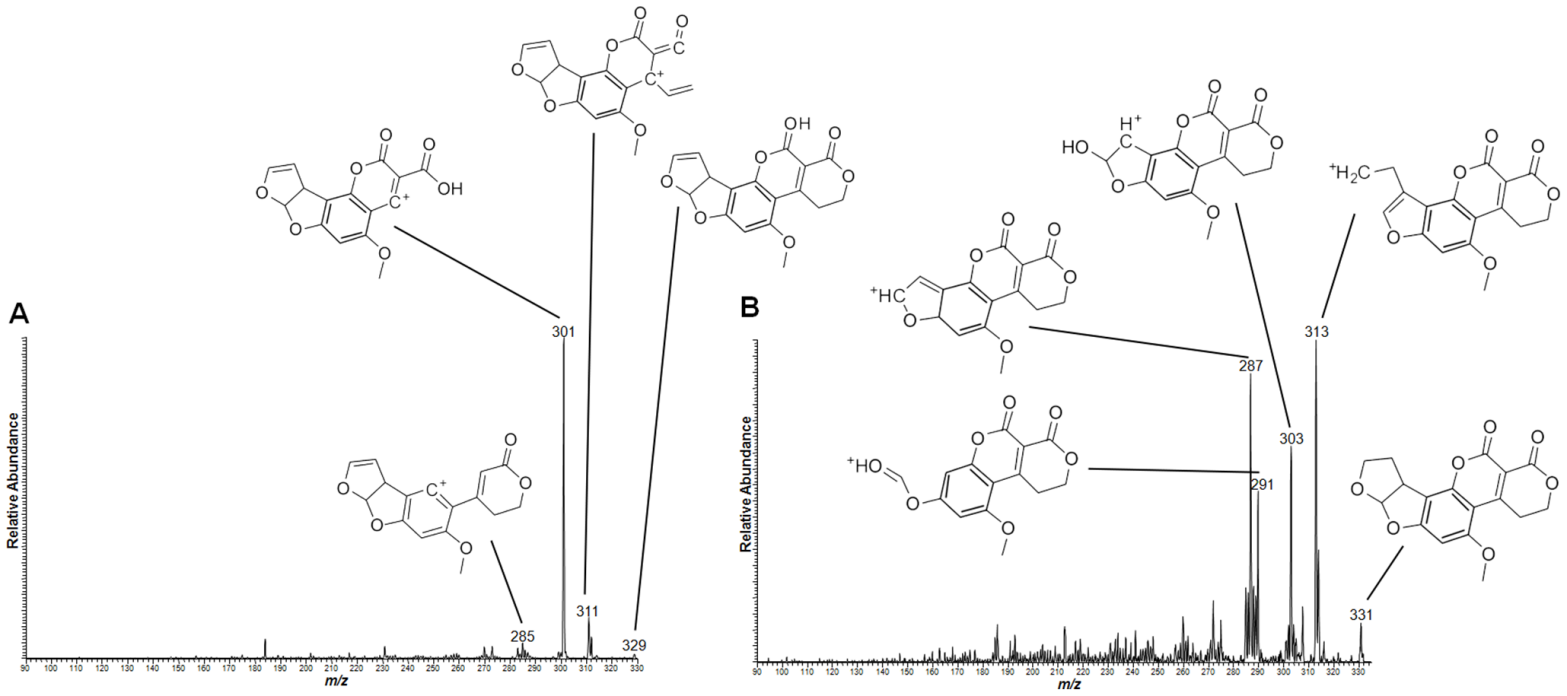
MS/MS spectra of aflatoxins (A) G1 and (B) G2. The characteristic fragments identified with Mass Frontier are identified along with the respective signals. Positive ion mode.

**Table 1:** Identified species and their CID products for structural elucidation.

Compound	Precursor ion → Product ions	
**Aflatoxins**	**[M+H]^+^**	**CID fragments**
	***m/z***	***m/z***
B1	313	295, 285, 271, 269, 255, 243
B2	315	297, 287, 271, 259, 254, 245
G1	329	311, 301, 285
G2	331	313, 303, 287, 291
**Stilbene**	**[M−H]^−^**	**CID fragments**
	***m/z***	**m/z**
Resveratrol	227	185, 159, 157, 145

**Figure 6:**
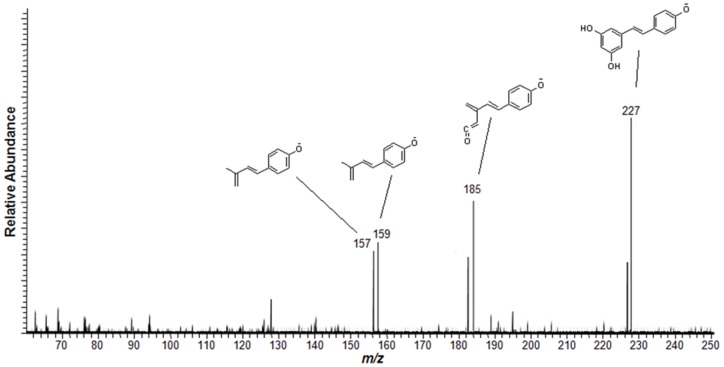
MS/MS spectrum of the compound identified as resveratrol at *m/z* 227 [M−H]^−^ and the characteristic product ions. Negative ion mode.

**Figure 7:**
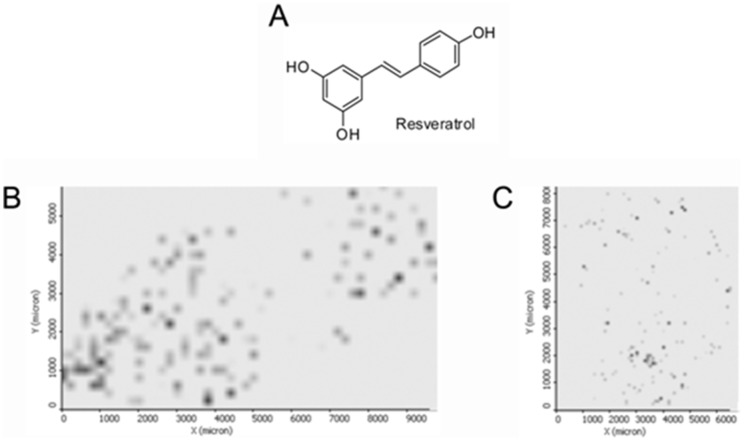
Schematic representation of the resveratrol molecule (A) and characteristic distribution on (B) peanut skin and (C) kernel. Data acquired in the negative ion mode.

MSI was also utilized to assess resveratrol, evaluating its spatial distribution using the same methodology as for the aflatoxins. The stilbenoid-derivative was also identified in the negative ion mode by MS/MS at resveratrol as [M−H]^−^ (*m/z* 227) with characteristic fragments, as elucidated in Figure 6. Figure 7 presents (A) the molecular structure of resveratrol and the spatial distribution of these compounds both in the (B) skin and (C) in the kernel of peanuts.

## Discussion

This novel approach on aflatoxin and phytoalexin detection directly on peanut surface has proven to be a fast and reproducible method. Without extensive sample preparation steps and no organic solvent employment, this technique shows great compromise with green chemistry trends [33]. Furthermore, this also avoids analyte losses in extraction and clean-up phases [34].

The use of MSI to identify both toxic and benefitial molecules in the same sample run is an effective and simpler approach. The possibility of identifying the colocalization of the targeted molecules directly from the sample surface is very interesting and may be appealing in terms of quality control and assurance. *Tandem* mass spectrometry also provides accurate structural information for the analyzed molecules, especially when compared to chemical standards. The use of MS/MS as the main identification tool for small molecules is largely described as a very useful and reliable approach [35], [36] and the use of a linear-trap quadrupole for these purposes is also feasible and compatible with this application, and is especially a well-established routine with MSI [37]–[40]. To the extent of this work, structural elucidation of the targeted molecules was supported by software-predicted molecular fragmentation. Mass Frontier is an expert system where CID products and fragmentation mechanisms can be modeled. To do so, it uses MS databases as well as algorithm calculations to propose fragmentation pathways and final product ions [41]. For this work, aflatoxin structures were proposed based on the matches between all obtained MS/MS experimental data and the calculated Mass Frontier fragments. To support even further the given information, structures of the product ions are presented in the sample spectra from Figures 4 and 5. For resveratrol, the same principles have been applied, and the results are plotted in Figure 6. This reinforces the high specificity of our methodology, where MS information is given with a high level of certainty.

Aflatoxin analysis is extremely relevant in terms of public health, as they are known for their carcinogenic effects and hepatotoxicity [42]. For the first time, the spatial distribution of these molecules is reported with information obtained directly from the skin and the kernel of peanuts, as illustrated by Figure 1. Interestingly, these mycotoxins present higher density towards the extremities of the skin and a more thorough distribution in the kernel. The analyzed phytoalexin, resveratrol, is a phenolic-derived compound. As well as an important role in plant defenses [10], this molecule is also important for human health and nutrition [43].

The overall amount of time dedicated to all analytical steps altogether (sample preparation, plate imprinting, instrumental analysis and data interpretation) can take as long as 15 minutes. This makes the presented approach a very fast and viable alternative for compound assessment directly from sample surface, with minimum sample preparation steps.

In summary, this work has demonstrated an effective analytical approach using SPILDI-MSI for direct assessment of aflatoxins and phytoalexins in peanut samples that has proven to be a simple and accurate strategy. This can be especially interesting for product treatment and toxin-removal processes, since it is possible to see that aflatoxins are not only present on the skin surface, but also in the more internal parts of the kernel.

## Supporting Information

Figure S1 **MS/MS spectra of the standard solutions of: (A) AFB1, (B) AFB2, (C) AFG1, (D) AFG2 and (E) resveratrol.** Aflatoxins were analyzed in the positive ion mode and resveratrol in the negative ion mode.
(TIF)
