# Supplementary figures and images for: Rapid and Simultaneous In Situ Assessment of Aflatoxins and Stilbenes Using Silica Plate Imprinting Mass Spectrometry Imaging
Source: PLoS One. 2014 Mar 4;9(3):e90901. doi: 10.1371/journal.pone.0090901 (PMC3942477; doi:10.1371/journal.pone.0090901)

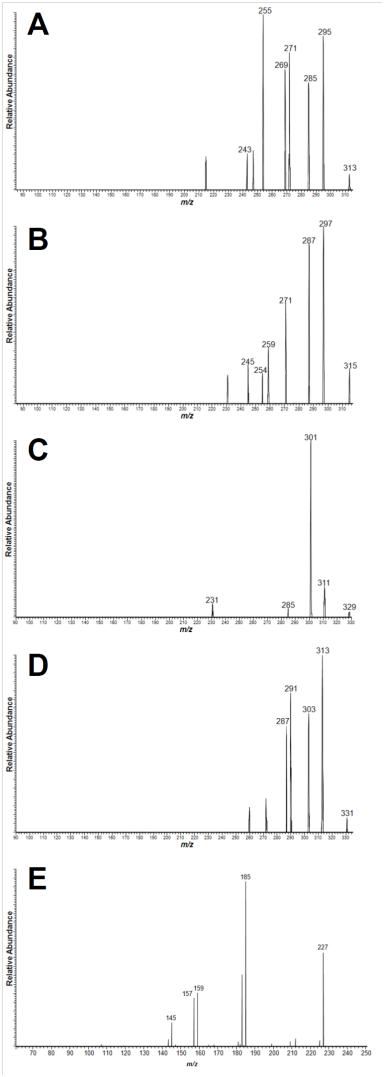

Supplement: Figure S1 — MS/MS spectra of the standard solutions of: (A) AFB1, (B) AFB2, (C) AFG1, (D) AFG2 and (E) resveratrol. Aflatoxins were analyzed in the positive ion mode and resveratrol in the negative ion mode. (TIF) [file pone.0090901.s001.tif]
